# Long-Term Survival in Patients with Oligometastatic Non-Small Cell Lung Cancer by a Multimodality Treatment—Comparison with Stage III Disease

**DOI:** 10.3390/cancers16061174

**Published:** 2024-03-17

**Authors:** Maja Guberina, Christoph Pöttgen, Nika Guberina, Christian Hoffmann, Marcel Wiesweg, Cedric Richlitzki, Martin Metzenmacher, Clemens Aigner, Servet Bölükbas, Thomas Gauler, Wilfried E. E. Eberhardt, Michael Forsting, Ken Herrmann, Dirk Theegarten, Kaid Darwiche, Verena Jendrossek, Martin Stuschke, Martin Schuler

**Affiliations:** 1 Department of Radiotherapy, West German Cancer Center, University Hospital Essen, University Duisburg-Essen, 45147 Essen, Germany; 2 National Center for Tumor Diseases (NCT) West, Campus Essen, 45147 Essen, Germany; 3 Department of Medical Oncology, West German Cancer Center, University Hospital Essen, University Duisburg-Essen, 45147 Essen, Germany; 4 Division of Thoracic Oncology, West German Cancer Center, University Medicine Essen—Ruhrlandklinik, 45239 Essen, Germany; 5 Department of Radiation Oncology, University Hospital, LMU Munich, 81377 Munich, Germany; 6 Department of Thoracic Surgery and Thoracic Endoscopy, West German Cancer Center, University Medicine Essen—Ruhrlandklinik, University Duisburg-Essen, 45239 Essen, Germany; 7 Department of Thoracic Surgery, Comprehensive Cancer Center, Medical University of Vienna, 1090 Vienna, Austria; 8 Institute of Diagnostic, Interventional Radiology and Neuroradiology, University Hospital Essen, University Duisburg-Essen, 45147 Essen, Germany; 9 Department of Nuclear Medicine, West German Cancer Center, University Hospital Essen, University Duisburg-Essen, 45147 Essen, Germany; 10 Institute of Pathology, West German Cancer Center, University Hospital Essen, University Duisburg-Essen, 45147 Essen, Germany; 11 Section of Interventional Pneumology, Department of Pulmonary Medicine, West German Cancer Center, University Medicine Essen—Ruhrlandklinik, University Duisburg-Essen, 45239 Essen, Germany; 12 Institute for Cell Biology (Cancer Research), University Hospital Essen, 45147 Essen, Germany

**Keywords:** non-small cell lung cancer, chemotherapy, immunotherapy, radiotherapy, stage III, oligometastatic disease

## Abstract

**Simple Summary:**

In the present work, we analyzed long-term outcomes of patients with synchronous oligometastatic (OMD) non-small cell lung cancer (NSCLC) with locally advanced tumors in comparison to patients treated for stage III NSCLC. All patients with oligometastatic NSCLC had to have a histopathologically confirmed diagnosis and were screened for driver mutations using next-generation sequencing. Patients with tumors showing EGFR, ALK or ROS1 gene alterations were excluded, and the PD-L1 tumor proportion score was determined. ^18^FDG-PET/CT and cranial MRI or CT were performed in all patients. All patients were treated with definitive or neoadjuvant radiochemotherapy plus surgery in addition to systemic therapy. Overall survival of OMD patients at five years was similar to that of stage III patients: 28.3% versus 34.9%. Baseline severe comorbidity, ECOG performance status, sex and pretreatment serum CRP level were the most important prognostic factors for the survival of OMD patients. Cumulative incidence of distant metastases was the highest competing risk for OMD patients, approaching 50% at 4 years. A multidisciplinary approach, including thoracic radiotherapy or a trimodality treatment, metastases-directed local therapy and contemporary systemic therapy, can lead to very good long-term survival of patients with oligometastatic and locally advanced non-small cell lung cancer, especially in patients without severe comorbidities and with good performance status.

**Abstract:**

**Background:** In patients with oligometastatic NSCLC, a cT3–cT4 primary tumor or an cN2/cN3 lymph node status was reported to be associated with unfavorable outcome. The aim of this study was to assess the importance of definitive or neoadjuvant thoracic radiochemotherapy for long-term outcome of these patients in order to find more appropriate treatment schedules. **Methods:** Analysis of the West Cancer Centre (WTZ) institutional database from 08/2016 to 08/2020 was performed. Patients with primary synchronous OMD, all without actionable driver mutations, who received definitive thoracic radiochemotherapy (RCT) or neoadjuvant RCT followed by surgery (trimodality treatment) were included. Survival outcome is compared with stage III NSCLC. **Results:** Altogether, 272 patients received concurrent radiochemotherapy. Of those, 220 presented with stage III (158 with definitive RCT, 62 with trimodality approach). A total of 52 patients had OMD patients with cT3/cT4 or cN2/cN3 tumors. Overall survival (OS) at five years for OMD patients was 28.3% (95%-CI: 16.4–41.5%), which was not significantly different from OS of patients with stage III NSCLC treated with definitive or neoadjuvant RCT (34.9% (95%-CI: 27.4–42.8%)). However, the PFS of OMD patients at five years or last follow-up was significantly worse than that of stage III patients (13.0% vs. 24.3%, *p* = 0.0048). The latter was due to a higher cumulative incidence of distant metastases in OMD patients (50.2% vs. 20.4% at 48 months, *p* < 0.0001) in comparison to stage III patients. A cross-validated classifier that included severe comorbidity, ECOG performance status, gender and pre-treatment serum CRP level as the most important factors in the univariable analysis, was able to divide the OMD patient group into two equally sized groups with a four-year survival rate of 49.4% in the good prognosis group and 9.9% in the poor prognosis group (*p* = 0.0021). Laboratory chemistry and clinical parameters, in addition to imaging and high-precision therapies, can help to predict and improve prognosis. **Conclusions:** A multimodality treatment approach and local metastases-directed therapy in addition to chemoimmunotherapy can lead to good long-term survival in patients with cT3/cT4 or cN2/cN3 OMD NSCLC without severe comorbidities and in good performance status and is therefore recommended.

## 1. Introduction

The hypothesis of the existence of an oligometastatic state assumes that in certain patients, distant metastases are restricted to a single or a few organs and that these patients may be amenable to a curative local therapy [1].

However, the time course of the cumulative incidences of loco-regional and distant relapses and the impact of loco-regional therapy is not sufficiently characterized.

There are mature data demonstrating that loco-regional thoracic radiotherapy can be safely conducted with concurrent chemotherapy and sequential immune checkpoint inhibition for larger thoracic target volumes, while data on concurrent chemo- and immunotherapy are just emerging [2,3,4].

In addition to stereotactic radiotherapy of brain metastases, stereotactic ablative radiotherapy of lung, liver and adrenal gland metastases has been demonstrated to be safe and effective [5,6,7,8].

According to current interdisciplinary guidelines, therapy recommendations for oligometastatic lung cancer take up an intermediate position between those for stages III and IV non-small cell lung cancer without actionable driver mutations on a rather limited empiric basis [9]. Petrelli et al. (2021) conducted a large meta-analysis in which a total of 104,234 patients (from 173 studies) were pooled. The OS was significantly better in the OMD group compared to more advanced tumors [10].

There are no restrictions in the authorization of immune checkpoint inhibitors in Europe for stage IV NSCLC without actionable driver mutations that limit their use in the oligometastatic state. The landscape of immunotherapeutic options has changed considerably over the last few years. Thus, there is a correlation that earlier studies with long-term follow-up were conducted with systemic therapies that have been overcome by new treatment options. This could particularly influence the simultaneous risk of distant and local recurrences [9,11,12].

In Germany, in 2019/2020 according to the Center for Cancer Registry Data, the overall survival rate across all lung carcinoma stages and histopathologic subgroups was 25% for women and 19% for men at 5 years. The 10-year survival rate was 19% and 14%, respectively [13,14].

In the metastatic stage, survival with conventional chemotherapy alone is less than 10% [15].

Several phase III trials showed improved survival by adding immune checkpoint inhibitors to first-line chemotherapy in stage IV NSCLC. Long-term survival outcomes of trials adding programmed death receptor-1 (PD-1) and programmed death ligand 1 (PD-L1) inhibitors such as atezolizumab, nivolumab, or pembrolizumab and nivolumab with or without the cytotoxic T-lymphocyte-associated protein 4 inhibitor (CTLA-4 inhibitor) ipilimumab, together with first-line chemotherapy, showed long-term survival at 4–5 years of 18–24% in comparison to 10–16% after chemotherapy alone [16,17,18,19,20]. Patients with tumors with high PD-L1 expression had a better 5-year survival of 23–30% [18,19]. Definitive thoracic surgery or radiotherapy for tumor control was not considered in these trials.

In this study, all consecutive patients with OMD and with local cT3/cT4 or cN2/cN3 NSCLC were included. All patients were treated in a recent time period from 08/2016 to 08/2020 with additional definitive radiochemotherapy or preoperative radiochemotherapy and surgery of the primary tumor, as well as involved mediastinal nodes.

The aim of the present study is to characterize the competing risk of relapse of patients with OMD NSCLC in comparison to patients with stage III NSCLC with long-term follow-up from the inclusion period, where immunotherapy was started but still infrequently used during induction chemotherapy for OMD NSCLC. In this time period, immunotherapy was routinely available as a component of second-line treatment. The second objective was to investigate whether, in the more homogeneous group of patients with OMD NSCLC with advanced cT- or cN categories, i.e., cT3/T4 or cN2/N3, the long-term prognosis at the endpoints of survival or incidence of distant metastases is independent or highly dependent on known patient- or treatment-related prognostic factors.

## 2. Materials and Methods

### 2.1. Study Design and Patients

The study was conducted at an academic lung cancer center and approved by the institutional ethics committee of the medical faculty [21-10033-BO]. This is a consecutive cohort study. The majority of the patients were enrolled in a prospective registry trial [18-8364-BO] from 03/2019.

Eligibility criteria included all consecutive patients with primary synchronous OMDLC (stage M1a/M1b/M1c (with a maximum 2–5 filiae in two differing organs); stage IVA/IVB (2–5 filiae) (according to the American Joint Committee on Cancer staging system, 8th edition)) [21] in the time interval from 08/2016 to 08/2020, who started with definitive or neoadjuvant concurrent radiochemotherapy of the primary tumor and involved mediastinal lymph nodes. Patients with metachronous or oligopersistent OMDLC were excluded (only inclusion of primary synchronous oligometastatic disease (ps-OMD)). All patients had to have a histopathologically confirmed diagnosis of NSCLC, and all tumors were screened for driver mutations using next-generation sequencing [22]. Patients with tumors with EGFR, ALK or ROS1 gene alterations were excluded. PD-L1 expression was determined using the BenchMar XT platform (by Roche Diagnostics, Ventana Medical Systems, Germany) and the anti-PD-L1 primary antibody 22C3. ^18^F-FDG-PET/CT and cranial MRI or CT were performed in all patients to define the accurate TNM stage [23].

Before the start of radiochemotherapy, all patients were discussed in an interdisciplinary tumor board. Patients who had started neoadjuvant radiochemotherapy were evaluated once more in the interdisciplinary tumor board to decide on the basis of an individual risk–benefit assessment whether to recommend resection or continuation of radiochemotherapy up to a definitive dose.

Surgical interventions or ablative radiochemotherapy of distant metastases were recorded.

Furthermore, the use of first-line chemoimmunotherapy before definitive radiochemotherapy, consolidation immunotherapy or the use of chemoimmunotherapy as second-line therapy was documented. Neoadjuvant chemotherapy was administered with two to three and, in selected cases, up to six cycles of platinum-based doublet regimen. Regular interim CTs were performed for response assessment. All patients were treated with intensity-modulated radiotherapy or volumetric-modulated arc radiotherapy. The clinical target volume (CTV) included the primary and affected lymph node stations according to EBUS-TBNA and ^18^F-FDG-PET/CT with a minimum margin of 5 mm or up to the anatomical borders. The prescription dose in the neoadjuvant setting was 45 Gy at 2 × 1.5 Gy per day on 5 days per week or 46.0 Gy at 1 × 2.0 Gy per day, and in the definitive treatment schedule, 60.0 Gy in conventional fractionation. Patients who received 45.0 Gy at 2 × 1.5 Gy per day in a neoadjuvant intent and who were deemed inoperable were treated with 65.0–71.0 Gy according to the results of the ESPATUE trial [24].

For comparison, a cohort of patients with stage III NSCLC with additional follow-up who were treated with definitive or neoadjuvant radiochemotherapy in a similar period from 01/2017 to 02/2020 at the same institution was considered [4]. In this group of patients, patients with tumors with known alterations in the EGFR, ALK or ROS DM were excluded.

All patients were followed up every 3 months for the first 2 years, every 6 months from years 2 to 5, and yearly thereafter.

### 2.2. Statistical Analysis

Ordinal or quantitative patient, tumor and treatment characteristics were described by their median as well as their range or interquartile range. Categorical characteristics from OMDLC and stage III patients were compared by the Fisher’s exact test and continuous data by the Wilcoxon two-sample tests. Overall survival (OS) and progression-free survival curves were calculated according to the Kaplan–Meier method. Endpoints were overall survival (OS) and progression-free survival (PFS), defined as the time between the start of radiotherapy, which is the date when the patient met the eligibility criteria, to the date of the considered event in order to avoid immortality bias. In addition, a competing risk analysis was performed using isolated loco-regional relapse, isolated distant relapse, combined local and distant relapse, and death without known relapse as mutually exclusive competing events. Statistical analyses were performed using SAS 15.1 (SAS Institute, Cary, NC, USA).

A leave-one-out cross-validated prognostic classifier with five parameters was built based on proportional hazard analysis and selection of the best parameter subset based on the chi-square (χ^2^-test) statistic of the largest global score [25]. The endpoint was overall survival. The i-th patient was classified as high-risk or low-risk depending on whether their predictive risk score according to the classifier from the leave-this-patient-out training dataset was higher, equal to or lower than the median value in the training set. This procedure was repeated for each patient.

Univariable proportional hazard analysis was performed for all patient-, tumor- or treatment-related characteristics. Characteristics with a prognostic value at *p* = 0.1 were selected for multivariable analysis. The validity of the proportional-hazards assumption was assessed by a Kolmogorov-type supremum test (PHREG procedure; SAS). The categorical characteristics were coded with 2-binary dummy variables. From these characteristics with importance in univariable analysis, the best cross-validated 5-parameter classifier was built.

In addition, propensity score weighting using logistic regression according to the standardized mortality ratio weighting approach was used to balance characteristics with known prognostic values between OMDLC and stage III NSCLC cohorts. Subjects’ weights were used to adjust the Kaplan–Meier survival estimates [26].

## 3. Results

### 3.1. Baseline Patient Characteristics

Table 1, Table 2 and Table 3 presents all patient characteristics of the 52 patients with OMDLC. All OMDLC patients had category cT3/4 primary tumors or cN2/N3 lymph node metastases. For comparison, patients with stage III disease treated in a similar time period at the same institution were included (Table 1, Table 2 and Table 3, Figure S1). Due to the presence of driver mutations (DMs), three patients in the OMDLC and two in the stage III cohort were excluded.

The median time from diagnosis by histopathology to start of radiotherapy was 4.1 months (range: 0.4–8.1 months).

Seven OMDLC patients were treated with neoadjuvant radiotherapy and received resection of the primary tumor and mediastinal lymph nodes. Patient-dependent characteristics did not differ between both groups. OMDLC patients had a smaller proportion of squamous cell carcinomas and tumors with high PDL-1 expression than stage III patients. The mean follow-up (FU) time of the OMDLC cohort amounts to 50.7 months, the interquartile range (IQR) 39.6–57.8 months, and that of the stage III cohort to 52.2 months (IQR 44.0–58.7 months), with no difference between the cohorts (*p* = 0.4497, log-rank test). Altogether, 43 OMDLC patients received solely induction chemotherapy, two combined neoadjuvant chemoimmunotherapy, five primary combined definitive radiochemotherapy, and three adjuvant immunotherapy. All OMD patients receiving definitive or neoadjuvant thoracic radiochemotherapy were candidates for metastases-directed therapy. Local therapy of synchronous distant metastases was performed in 16 patients with surgery and in 29 patients with radiotherapy during first-line therapy. Stereotactic ablative radiotherapy of brain or lung metastases was performed in 16 of the latter patients, while the remaining received hypofractionated metastases-directed radiotherapy with a biologically effective dose of at least 50 Gy, assuming a repair capacity of alpha/beta that equals 10 Gy. The seven patients not receiving definitive local therapy to the synchronous distant metastases showed either early progression after definitive radiochemotherapy or were in complete remission at the distant site. Of all OMDLC patients, 29 required a second-line treatment, including immunotherapy. Overall, 38.9% (7/18) of all OMDLC patients who did not receive immunotherapy were in complete remission at the last follow-up.

### 3.2. OMD Patients

The diagnosis was confirmed in all OMD patients by EBUS-TBNA, PET/CT and brain MRI staging. In the selected cohort of primary synchronous oligometastatic disease (ps-OMD), nearly all (86.5%) had metastases in only one organ site at treatment start and presented with a single metastasis (75.0%). The others showed less than five metastases in one or two organs. Twelve patients had pulmonary metastases, eleven brain metastases, seven osseous metastases, and six patients had adrenal metastases, five of which were histologically confirmed by biopsy or resection. Three patients had pleural metastases, three patients had metastases in distant lymph nodes and three in other locations. Seven patients had metastases in two organ systems. Regarding thoracic tumor spread of OMDLC patients, 94.3% of OMDLC patients had locally advanced disease, a T and N combination corresponding to stage III, while ignoring the M category. Forty-five of the OMDLC patients received definitive radiochemotherapy for thoracic tumor spread, and seven received multimodality therapy, including surgery. Altogether, 86.5% of all ps-OMD patients received a definitive treatment (resection or radiotherapy) of the metastases. The laboratory parameters hemoglobin, C-reactive protein (CRP) and lactate dehydrogenase (LDH) at the start of treatment were 13.4 g/dL (9.4–16.2), 1.50 mg/dL (0.0–13.8) and 217 U/L (99–398), respectively, and at the end of radiotherapy, 11.2 (7.5–15.2), 0.9 mg/dL (0–6.8) and 237 U/L (124–888), respectively.

### 3.3. Stage III Patients

**Figure 1:**
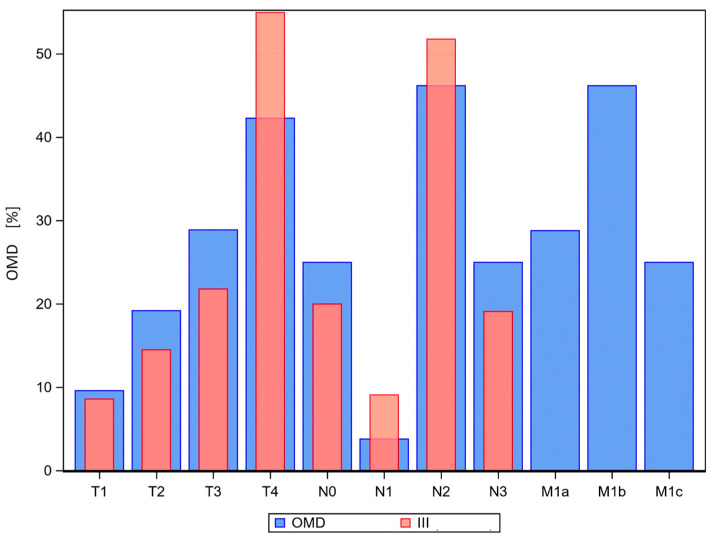
Distribution of the TNM classifiers in the respective cohort of patients with NSCLC III and OMD; NSCLC OMD blue columns, NSCLC stage III red columns.

At stage III diagnosis, there were 220 patients. Of all, 55% presented with cT4 disease. Altogether, 158 were classified as functionally and technically inoperable. The median age was 64.9 years (Figure 1).

### 3.4. Overall Survival and Progression-Free Survival

**Figure 2:**
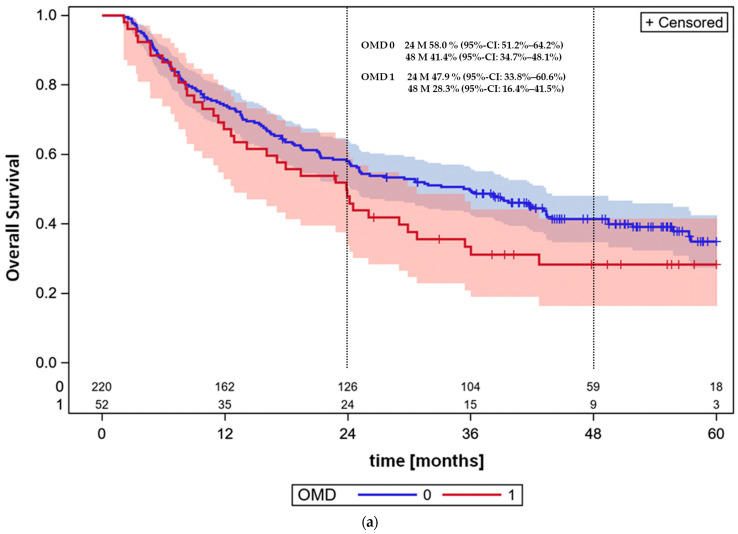

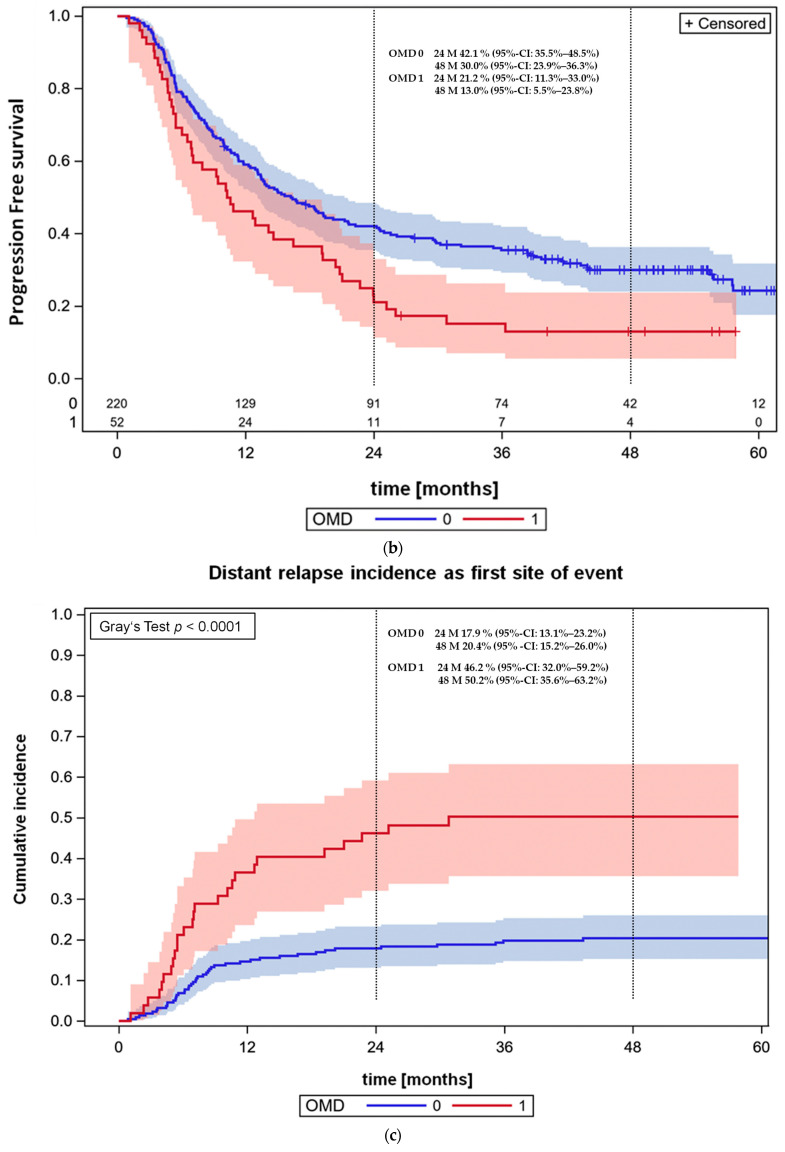

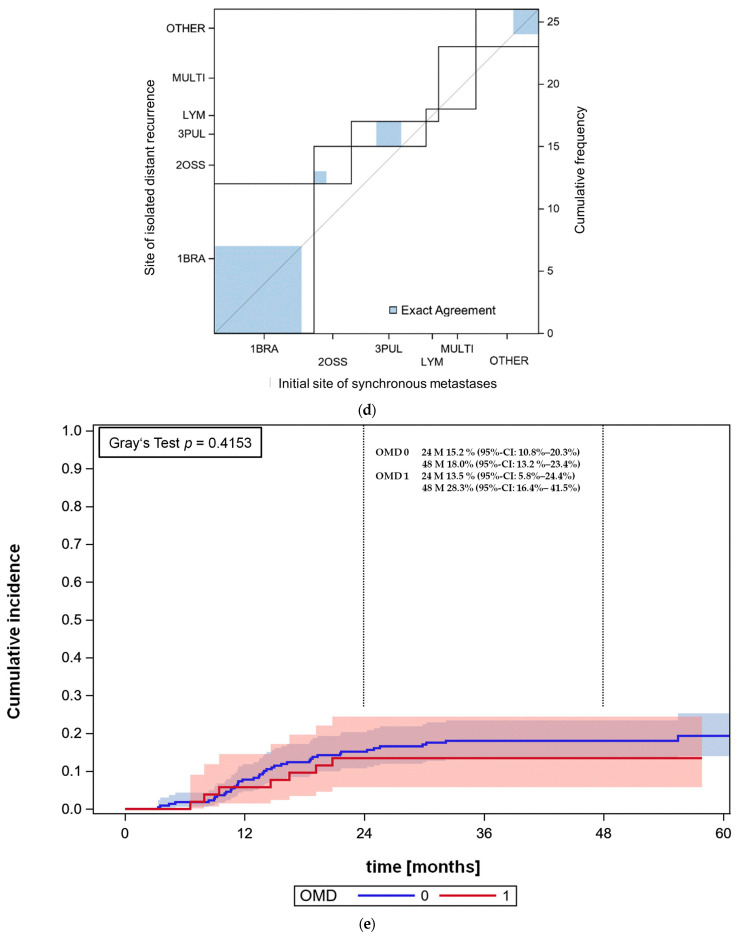

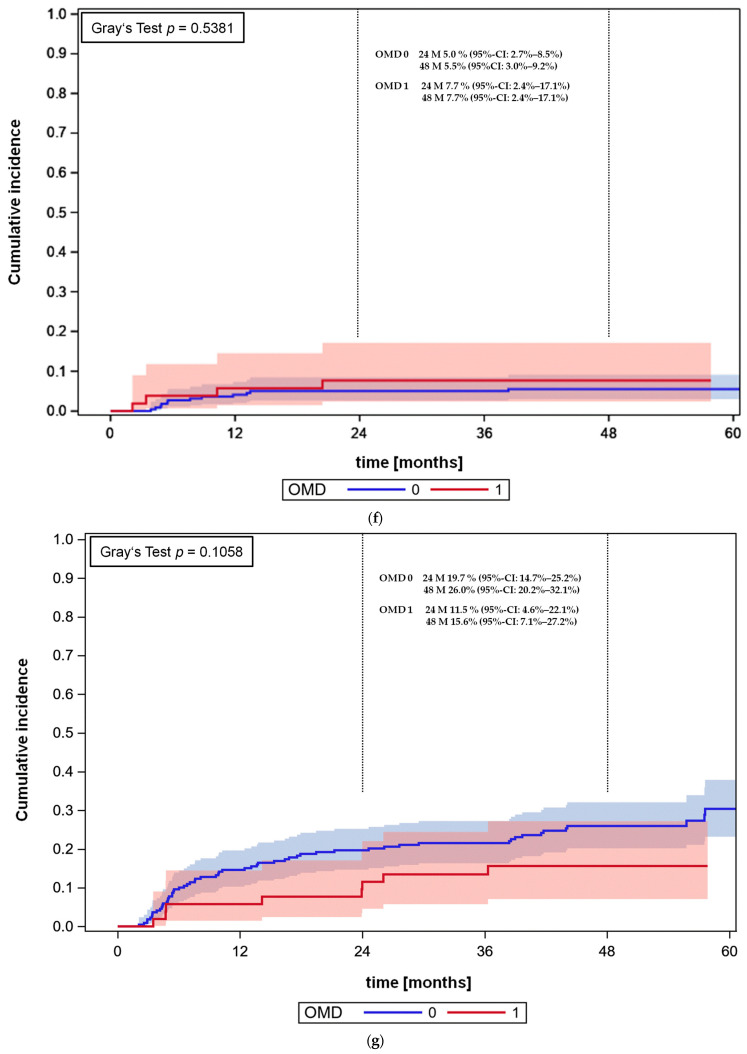
**OMD = 0: In this cohort all stage III patients; OMD = 1: OMD patients.** (**a**) Overall survival, whole cohort sampled by AJCC stage III NSCLC (N = 220) vs. OMD NSCLC patients (N = 52) without driver mutations (DMs). The survival was not significantly different (*p* = 0.1122, logrank test). Follow-up time is presented in months. (**b**) Progression-free survival of stage III and OMD patients (*p* = 0.0048, log-rank test). (**c**) Distant relapse incidence as first site of event. OMD patients had 26 events and 19 concurrent events, stage III patients 44 events and 110 concurrent events. The cumulative incidence of distant recurrences as first event was 50.2% (95%-CI: 35.6–63.2%) at 48 months for OMD patients and 20.4% (95%-CI: 15.2–26%) for stage III patients, *p* < 0.0001, Gray’s test. (**d**) Agreement plot: Sites of distant metastases at initial diagnosis of synchronous OMDLC and at the time of recurrence as first site of relapse. Agreement plot as a visual representation of a contingency table displays the sites of distant metastases at the initial diagnosis of synchronous OMDLC and at the time of recurrence for the 26 patients with distant recurrences as first site of relapse. The frequencies of patients with metastases in the different sites are shown at diagnosis (*x*-axis) and at the time of relapse (*y*-axis). Shaded boxes (exact agreement) indicate the frequencies of patients with metastases in the same site at the different time points. (**e**) Loco-regional relapse as first event, OMD: N = 52, 7 events at 38 concurrent events, cumulative incidence of distant recurrences as first event at 48 M for OMD patients: 13.5% (95%-CI: 5.8–24.4%); stage III patients, N = 220, 40 events at 114 concurrent events, cumulative incidence at 48 M: 18.0% (95%-CI: 13.2–23.4%), Gray’s test, *p* = 0.4153. (**f**) Combined loco-regional and distant relapses as first event. OMD: N = 52, 4 events at 41 concurrent events, cumulative incidence of combined recurrences as first event at 48 M for OMD patients: 7.7% (95%-CI: 2.4–17.1%); stage III patients: N = 220, 12 events at 142 concurrent events, cumulative incidence at 48 months: 5.5% (95%-CI: 3.0–9.1%), Gray’s test for difference between cumulative incidences, *p* = 0.5381. (**g**) Death without known relapse at any site, OMD: N = 52 pts, 8 events at 37 concurrent events, cumulative incidence of deaths without known recurrence at 48 M for OMD patients: 15.6% (95%-CI: 7.1–27.2%); stage III patients, N = 220, 58 events at 96 concurrent events. Cumulative incidence at 48 M: 25.9% (95%-CI: 20.2–32.1%). Cumulative incidence curves were not significantly different (*p* = 0.1058, Gray’s test).

The 5y-OS of OMD patients was 28.3% (95%-CI: 16.4–41.5%) and 34.9% (95%-CI: 27.4–42.8%) for the consecutive patients with stage III NSCLC treated in a similar time period at the same institution with definitive or neoadjuvant radiochemotherapy (Figure 2a). The difference between both cohorts was not significant (*p* = 0.1122, log-rank test). The hazard ratio of death of stage III in comparison to the OMDLC cohort was 0.74 (95%-CI: 0.51–1.07).

Figure 2b shows progression-free survival rates in the OMDLC and stage III cohorts. Progression-free survival at 48 months was 13.0% (95%-CI: 5.5–23.8%) in the OMDLC cohort and 24.3% (95%-CI: 17.5–31.7%) at 60 months in the stage III cohort. The difference between both curves was significant (*p* = 0.0048, log rank test). The hazard ratio at this endpoint was 0.62% (95%-CI: 0.45–0.87%).

Figure 2c,e–g show the results of the competing risks analysis. Figure 2c depicts the distant relapse rates as first site of event in the OMDLC NSCLC cohort compared to the stage III NSCLC cohort. Distant relapse rates were significantly higher in the OMDLC cohort (*p* < 0.0001). Figure 2d–f demonstrate loco-regional relapse rates as first event, combined loco-regional and distant relapses as first event and death rates without known relapse at any site in all patients.

To further analyze the importance of loco-regional and distant recurrences, a competing risk analysis was performed. The cumulative incidence of distant recurrences as first event was 50.2% (95%-CI: 35.6–63.2%) at 48 months for OMDLC patients and 20.4% (95%-CI 15.2–26.0%) for stage III patients. The difference was significant between groups (*p* < 0.0001, Gray’s test; Figure 2c). There was evidence that the site of distant relapses agreed with the site of synchronous initial metastases in the 26 patients with distant relapses as first site of first relapse (Figure 2d). The kappa coefficient, together with its standard error, was 0.3106 ± 0.0924 and, therefore, differed from 0 (*p* = 0.0008, two-sided z-test), indicating that the agreement was not purely by chance.

In contrast to distant metastases, the cumulative incidence of loco-regional relapses as first event was similar between OMDLC and stage III patients, with 13.5% (95%-CI: 5.8–24.4%) and 18.0% (95%-CI: 13.2–23.4%) at 48 months (*p* = 0.4153, Gray’s test, Figure 2e). Therefore, the higher incidence of distant relapses was the main cause for the difference in progression-free survival between OMDLC and stage III NSCLC patients.

Combined loco-regional and distant relapses as first event were rare in both OMDLC and stage III patients, consistent with an independent occurrence of distant and loco-regional relapses. The cumulative incidence of combined recurrences was 7.7% (95%-CI: 2.4–17.1%) at 48 months for OMDLC patients and 5.5% (95%-CI: 3.0–9.1%) for stage III patients (*p* = 0.5381, Gray’s test, Figure 2f).

The cumulative incidence of death without known relapse at any site was 15.6% (95%-CI: 7.1–27.2%) at 48 months for OMDLC and 25.9% for stage III patients (*p* = 0.1058, Gray’s test). As OMDLC patients have a higher risk of distant relapses, this concurrent risk can result in a lower incidence of deaths without known relapse at any site, given the same underlying risks of intercurrent deaths in both cohorts without consideration of other types of events.

### 3.5. Cross-Validated Prognostic Survival Classifier for the OMD Patient Cohort

**Figure 3:**
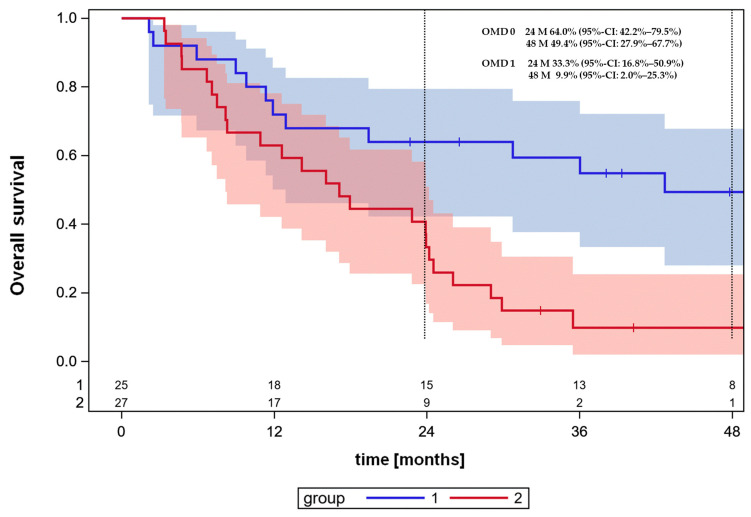
Leave-one-out cross-validated Kaplan–Meier survival curves for the high- and low-risk groups of OMD patients according to the prediction model built from the training sets, according to patient’s linear 5-parameter predictor that is larger, equal or smaller than the median in the training set. The selected prognostic parameters were severe comorbidity, CRP serum level at the time of diagnosis, sex and ECOG status. ps-OMD: primary synchronous oligometastatic disease.

The association of all patient-, tumor- or treatment-dependent characteristics given in Table 1, Table 2 and Table 3 was analyzed by univariable proportional hazards analysis for the OMD cohort. Factors that become important with a *p*-value <0.05 were sex (*p* = 0.0198), severe comorbidity (*p* = 0.0214), serum C-reactive protein level at diagnosis (*p* = 0.0063, χ^2^-test) and ECOG status at the start of radiochemotherapy (*p* = 0.0491, χ^2^-test). The categorical variable ECOG with three possible values was coded with 2-binary dummy variables. From these characteristics, which were of significance in univariable analysis, the cross-validated 5-parameter classifier was built. The cross-validated survival curves, according to the 5-parameter classifier, are shown in Figure 3, with significant differences between the high- and low-risk groups (*p* = 0.0021, log-rank test). Good prognosis patients, i.e., patients with good ECOG performance status without severe comorbidity, with low CRP values and metastases-directed local therapy, had a very good survival of 49.4% (95%-CI: 27.9–67.7%) at 48 months in comparison to the poor prognosis patients of 9.9% (95%-CI: 2.0–25.3%) (*p* = 0.0021, log-rank test). The hazard ratio of the low-risk in comparison to the high-risk group was 2.965 (95%-CI: 1.443–6.095). The cumulative incidences of distant metastases as isolated first site of relapse that differed in the high- and low-risk groups were not different.

### 3.6. Propensity Score Weighted Survival Analysis: Age, CRP at Diagnosis, Sex, Severe Comorbidity

**Figure 4:**
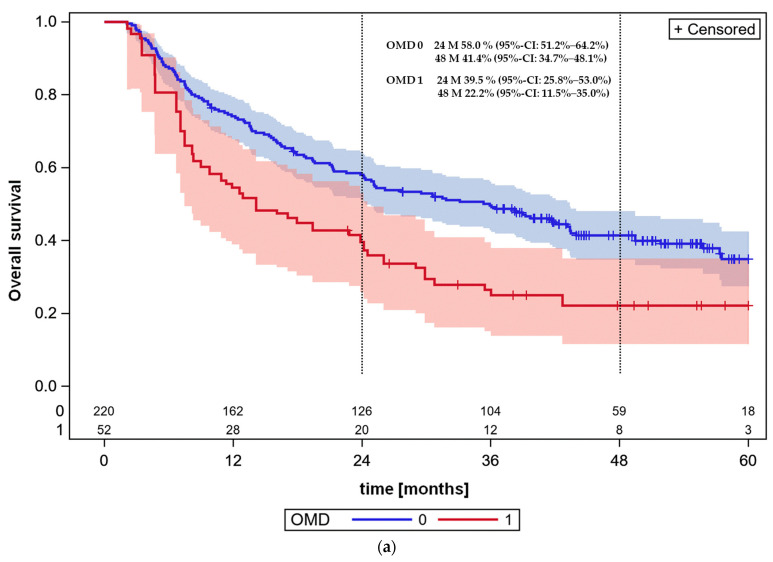

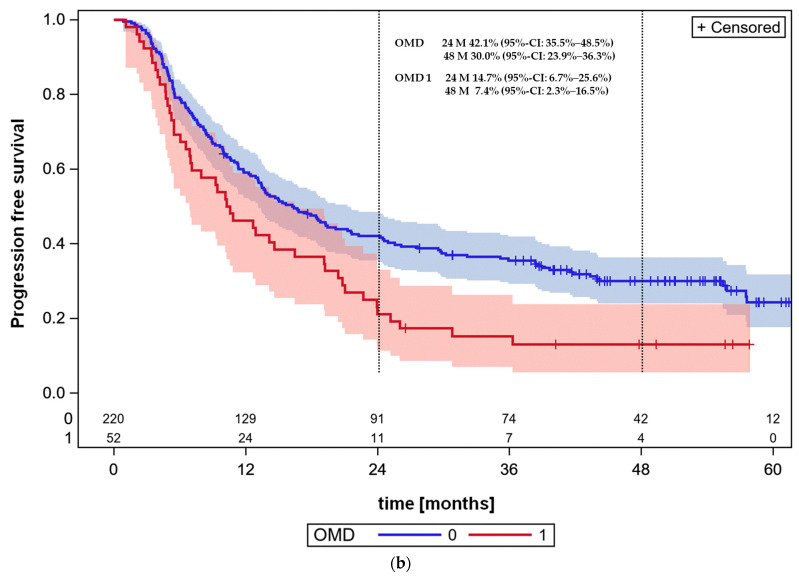
(**a**) Overall survival in the OMD and stage III NSCLC patient cohorts using propensity score weights (*p* = 0.0036, log-rank test). OMD = 0: stage III patients; OMD = 1: OMD patients. (**b**) Propensity score weighting, progression-free survival, N = 272, all OMD patients without DMs, ps-OMD 12.7% (5.4–23.3), *p* = 0.0030. Not-OMD = stage III = 0; OMD = 1.

The survival curves of the OMDLC and stage III NSCLC cohorts were additionally compared after propensity score weighting of the individuals. The characteristics included in estimation of propensity score weights were those that become important in univariable proportional hazards survival analysis of OMD patients, i.e., sex, CRP at diagnosis, ECOG status and the presence of severe comorbidities (Table 4). Using propensity score weighting, survival was found to be inferior in the OMDLC cohort compared to the stage III NSCLC patient cohort (*p* = 0.0036, log-rank test, Figure 4a). The adjusted Kaplan–Meier estimate of overall survival at 60 months was 22.2% (95%-CI: 11.5–35.0%) for the OMDLC and remained unchanged at 34.9% (95%-CI: 27.4–42.8%) for the stage III NSCLC patients using standardized mortality ratio propensity score weighting. The results of propensity score weighting underline the great influence of the prognostic factors on outcome (Figure 4a,b). The progression-free survival curves for the stage III and OMD patients, using propensity score weighting, are shown in Figure 4b with significant difference between curves (*p* < 0.0001, log-rank test). The adjusted Kaplan–Meier estimates of progression-free survival at 48 months or last follow-up were 7.3% (95%-CI: 2.2–16.5%) for the OMDLC and 24.3% (95%-CI: 17.5–31.7%) at 5 years for the stage III NSCLC patients.

## 4. Discussion

In this study, we analyzed the long-term outcome of a consecutive and larger OMD cohort at a lung cancer center and the main prognostic parameters affecting survival improvement in recent years.

Local therapies of the primary tumor and distant metastases is a recommended option for synchronous OMD NSCLC patients in current interdisciplinary and radiation oncological guidelines [9,12].

Recently published randomized trials on synchronous OMD in NSCLC lung cancer patients had moderate follow-up and small sample sizes, with 25 or fewer patients per treatment arm, and were conducted prior to the introduction of immunotherapy in stage IV lung cancer [12,27]. Median PFS rates were 9.7 months and 14.2 months in the treatment arms with local consolidative therapy. This was significantly better than those without consolidative therapy. In the trial by Gomez et al., one long-term survivor at 5 years was observed [27]. In the trial of Wang et al. on EGFR-mutated oligometastatic NSCLC without brain metastases, radiotherapy of the primary tumor and the singular metastases in addition to a first-line tyrosine-kinase inhibitor significantly prolonged overall survival to 36 months [28].

There are several single-arm prospective trials on NSCLC patients with synchronous OMD [29,30,31]. The trial of Arrieta et al. showed promising long-term survival after local therapy of the primary tumor and metastatic sites, with overall survival rates of about 30% at 4 years [30,31].

Other larger retrospective trials found the cT3/cT4 and cN2/cN3 categories as unfavorable prognostic factors for NSCLC patients with OMD [32,33,34,35,36,37].

Here, we could not confirm this statement, especially not for patients with an ECOG performance status of 0 and without severe comorbidities.

Several retrospective studies also analyzed prognostic factors in OMD patients. A reduced performance status, comorbidity and sex were identified as poor prognostic factors in other studies as well as in the present study [37,38,39]. Negative prognostic factors such as poorer performance status and severe comorbidity did not only influence prognosis but could also decrease treatment tolerance [40]. Other study groups have also found CRP serum levels to be an unfavorable prognostic factor for NSCLC patients [41].

In this study, propensity score weighting according to the above prognostic factors tended to decrease survival of OMD patients in comparison to stage III patients. This was predominately due to the higher prevalence of severe comorbidities in stage III patients, which was numerically higher in stage III than in OMD patients. Therefore, propensity score weighting led to a higher weight of these worse prognosis patients in OMD patients. (Figure S2)

Age was comparable in our stage III and OMD cohort and was not a prognostic factor in this study. In accordance with this, other studies imply that frailty, performance status, and comorbidity are of greater importance than chronological age itself [42].

Patterns of relapse after local consolidation of synchronous OMD NSCLC patients were reported by a few studies, identifying distant relapses as a major site of relapse in over 50% of patients [33,37,38,43]. According to the present study, distant relapses were the dominant concurrent risk in this study, with a cumulative incidence of 51.2% at 48 months, which was about 2.5 times higher than in stage III NSCLC patients treated with definitive or neoadjuvant radiochemotherapy. There was a moderate concordance of organ location of distant relapses after first-line treatment and the synchronous metastatic sites. However, in half of the patients with a good prognosis, according to the cross-validated classifier, the incidence of new distant metastases was only moderate at 37% at 48 months. Immunotherapy in combination with first-line chemotherapy [16,17,18,19,20] has shown consistent benefit in stage IV adeno- and squamous cell carcinomas in addition to chemotherapy [9] and is an integral part of recent ongoing prospective trials for OMD NSCLC patients [44]. In the present study, about 10% of patients received immunotherapy during the first-line chemotherapy phase, while the majority of patients received immunotherapy in later lines of treatment. This is a limitation of this study, due to the inclusion period, as the use of immunotherapy in combination with first-line chemotherapy has steadily increased. However, unlike earlier randomized trials on local therapy in OMD patients, immunotherapy was used in most of the OMD patients in this study as second-line treatment after progression. The main columns in this cohort were a thorough initial staging, including ^18^F-FDG-PET/CT, EBUS-TBNA and cranial MR, as well as new therapeutic options such as precision radiation techniques and microinvasive surgical and standard combination systemic therapy options.

Studies on the long-term outcomes of OMD NSCLC patients treated in the period between 2016 and 2020, as in the present study, are rather scant. Given the risk of false-positive PET/CT findings [45,46,47], studies with histopathological verification of distant metastases are of great value. In this study, about 30% of patients received surgery for their distant metastases, and the majority of adrenal gland metastases were histopathologically proven.

Definitive thoracic radiochemotherapy or trimodality therapy, including surgery of the primary tumor and nodal metastases, as well as local metastases-directed therapy in addition to chemoimmunotherapy, can lead to good long-term survival of cT3/cT4 or cN2/cN3 OMD NSCLC patients without severe comorbidities and with good ECOG performance status and, therefore, can be recommended.

## 5. Conclusions

A multidisciplinary treatment approach in oligometastatic and locally advanced non-small cell lung cancer, including thoracic radiotherapy for tumor control, can lead to favorable overall and progression-free survival and even allow a sustained and complete tumor response.

## Figures and Tables

**Table 1:** Total cohort: Patient characteristics (Primary set-up, N = 272).

Patient, Tumor and Treatment Characteristics	Stage III (N [%])	OMD (N [%])	*p*-Value
**Age** in years: median (range) **Age groups:** <40 y/40–≤60 y/60–≤70 y/>70 y	64.9 (43.6–87.0) 0/65/85/70 [0.0/29.5/38.6/31.8]	63.8 (38.7–87.8) 1/16/23/12 [1.9/30.8/44.2/23.1]	0.1060
**Sex:** male/female	150/70 [68.2/31.8]	33/19 [63.5/36.5]	0.6252
**T-category:** T1/T2/T3/T4	19/32/48/121 [8.6/14.5/21.8/55.0]	5/10/15/22 [9.6/19.2/28.9/42.3]	0.3762
**N-category:** N0/N1/N2/N3	44/20/114/42 [20.0/9.1/51.8/19.1]	13/2/24/13 [25.0/3.8/46.2/25.0]	0.4183
**M-category:** M0/M1a/M1b/M1c	220/0/0/0 [100/0/0/0]	0/15/24/13 [0/28.8/46.2/25]	**<0.0001**
**UICC Stage:** IIIA/IIIB/IIIC/IVA/IVB	100/91/29/0/0 [45.4/41.4/13.2/0/0]	0/0/0/44/8 [0/0/0/84.6/15.4]	**<0.0001**
**Thoracic stage, ignoring M-category:** **IIB/IIIA/IIIB/IIIC**	0/100/91/29 [0/45.4/41.4/13.2]	3/21/21/7 [5.8/40.4/40.4/13.5]	**0.0249**
**ECOG performance status:** 0/1/2/3	99/92/29/0 [45/41.8/13.2/0]	25/21/6/0 [48.1/40.4/11.5/0]	0.9308
**Pre-existing comorbidity** classified as grade > 2 according to CTC-AE-version 5: yes vs. no	63/157 [28.6/71.4]	9/43 [17.3/82.7]	0.1161
**Histology:** squamous/non-squamous	113/107 [51.4/48.6]	13/39 [25.0/75.0]	**0.0006**
**COPD GOLD:** 0–I/II/III/IV (%)	85/101/27/7 [38.6/45.9/12.3/3.2]	27/21/4/0 [51.9/40.4/7.7/0]	0.2680
**PD-L1 expression:** 0%/1%–<50%/≥50%/(n/d)	69/63/27/61 [31.4/28.6/12.3/27.7]	27/10/9/6 [51.9/19.2/17.3/11.5]	**0.0076**
**^18^F-FDG-PET/CT before therapy:** no/yes	18/202 [8.2/91.8]	0/52 [0/100]	**0.0289**
**EBUS-TBNA before therapy:** no/yes	17/203 [7.7/92.3]	4/48 [7.7/92.3]	1.0000

Continuous variables were compared by Wilcoxon two-sample test, nominal variables were compared by Fisher’s exact test. All numbers represent counts of patients in stage III or OMD groups for categorical data. COPD GOLD: chronic obstructive pulmonary disease Global Initiative for Chronic Obstructive Lung Disease; CRP: C-reactive protein; CTC-AE: Common Terminology Criteria for Adverse Events; EBUS-TBNA: endobronchial ultrasound-guided transbronchial needle aspiration; ECOG: Eastern Cooperative Oncology Group; IQR: interquartile range; n/d: not determined; TNM category: staging according to primary tumor extension (T), nodal (N) and distant metastases (M); UICC: Union for International Cancer Control; in square brackets [%], in round brackets (ranges).

**Table 2:** Patient characteristics: Laboratory data set (Primary set-up, N = 272).

Patient, Tumor and Treatment Characteristics	Stage III (N [%])	OMD (N [%])	*p*-Value
**Hemoglobin at diagnosis** in g/dL: median (range)	13.5 (7.8–17.9)	13.4 (9.4–16.2)	0.9885
**LDH at diagnosis** in U/L: median (range)	218 (114–1116)	217 (99–398)	0.6869
**CRP at diagnosis** in mg/dL: median (range)	1.45 (0.0–25.0)	1.50 (0.0–13.8)	0.5041

Continuous variables were compared by Wilcoxon two-sample test, nominal variables were compared by Fisher’s exact test. All numbers represent counts of patients in stage III or OMD groups for categorical data. LDH: lactate dehydrogenase; CRP: C-reactive protein; in square brackets [%], in round brackets (ranges).

**Table 3:** Patient characteristics: Treatment (Primary set-up, N = 272).

Patient, Tumor and Treatment Characteristics	Stage III (N [%])	OMD (N [%])	*p*-Value
**Treatment modality:** **Definitive radiochemotherapy/Trimodality treatment (%)**	158/62 [71.8/21.2]	45/7 [86.5/13.5]	**0.0328**
**Total radiation dose in Gy**: Median (interquartile range)	61.0 (47.0–65.5)	60.0 (60.0–64.0)	0.5372
**Date of Start of radiotherapy:** **Days from 1 August 2016,** **Median (range)**	687 (17–1286)	707 (158–1556)	0.7555
**ICI Line:** none (0)/1/2/3	124/38/54/4 [56.4/17.3/24.5/1.8]	18/5/26/3 [34.6/9.6/50.0/5.8]	**0.0007**
**Therapy of synchronous distant metastases:** **systemic therapy (ST) alone/ST + RT/ST + surgery**	Not applicable	7/29/16 [13.5/55.8/30.8]	Not applicable

Continuous variables were compared by Wilcoxon two-sample test, nominal variables were compared by Fisher’s exact test. All numbers represent counts of patients in stage III or OMD groups for categorical data. Gy: Gray (unit); ICI line 0–4: immune checkpoint inhibitor/treatment line: 0 = no ICI applied, 1 = first-line, 2 = second-line, 3 = third-line; RT: radiotherapy, ST: systemic therapy; in square brackets [%], in round brackets (ranges).

**Table 4:** Survival analyses: Known prognostic parameters used for propensity score weighting.

	HR (95%-CI (Range))	*p*-Value
Sex: female vs. male	0.405 (0.190–0.866)	0.0198
Severe comorbidity	2.552 (1.149–5.669)	0.0214
C-reactive protein *	1.130 (1.035–1.234)	0.0063
ECOG status **	2.102 (1.057–4.179)	0.0342

Important prognostic parameters for survival according to univariable proportional hazards analysis; sex: female vs. male, severe comorbidity, * C-reactive protein serum levels per unit increase, ** ECOG performance status, ECOG 0 versus ECOG1/2.

## Data Availability

Part of the data is available and can be received by the corresponding author after definition of the secondary trial analyses.

## Supplementary Materials

The following supporting information can be downloaded at: https://www.mdpi.com/article/10.3390/cancers16061174/s1, Figure S1: Flow chart, including of all patients receiving definitive and trimodality treatment. Figure S2: Overall Survival in female and male patients, Overall Survival dependent on ECOG performance status and Overall Survival dependent on presence of severe comorbidity.
