# Supplementary material for: Long-Term Survival in Patients with Oligometastatic Non-Small Cell Lung Cancer by a Multimodality Treatment—Comparison with Stage III Disease
Source: Cancers (Basel). 2024 Mar 17;16(6):1174. doi: 10.3390/cancers16061174 (PMC10969158; doi:10.3390/cancers16061174)
Supplement: Supplementary file 1 [file cancers-16-01174-s001.zip › cancers-2884468-supplementary.pdf]

Supplement Figures:

Supplementary Materials: Figure 1 Supplement: Flow chart, including of all patients receiving definitive and trimodality treatment, OMD Non-small cell lung cancer patients = OMDLC patients: N = 45 + 7 = 52.

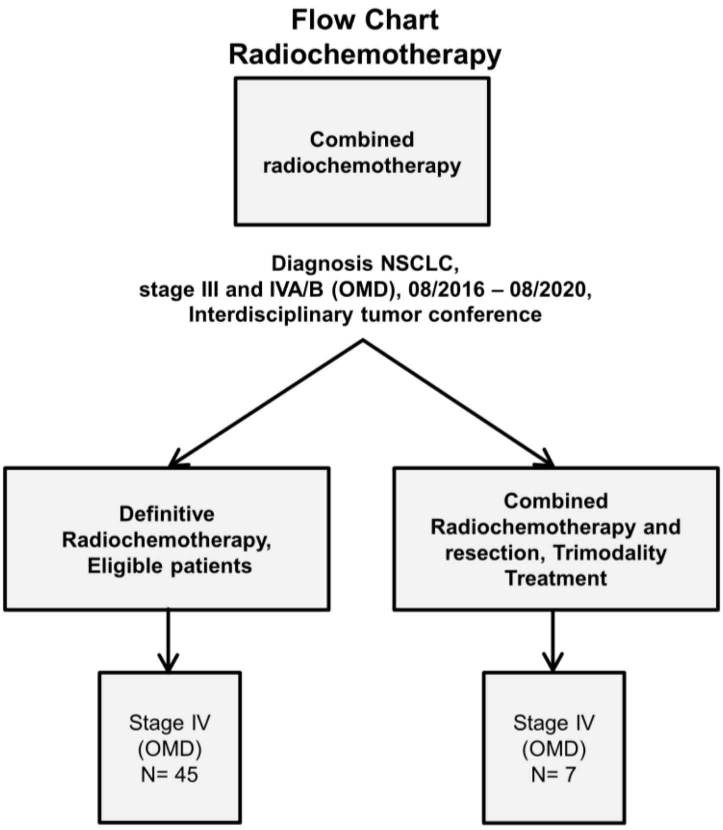

Figure S1: Flow chart, including of all patients receiving definitive and trimodality treatment, OMD Non-small cell lung cancer patients = OMDLC patients: N = 45 + 7 = 52.

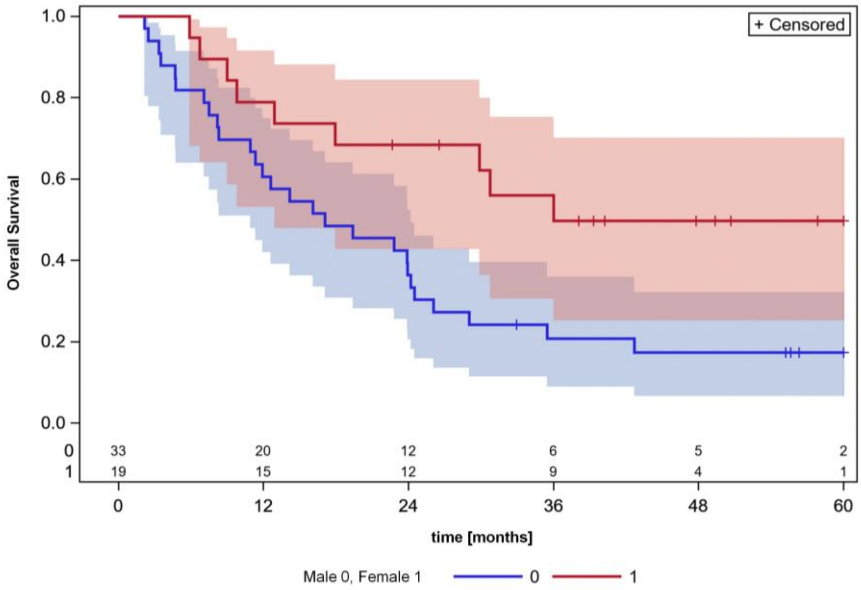

Figure S2 (a): Overall Survival in female [red line 1] and male patients [blue line 0].

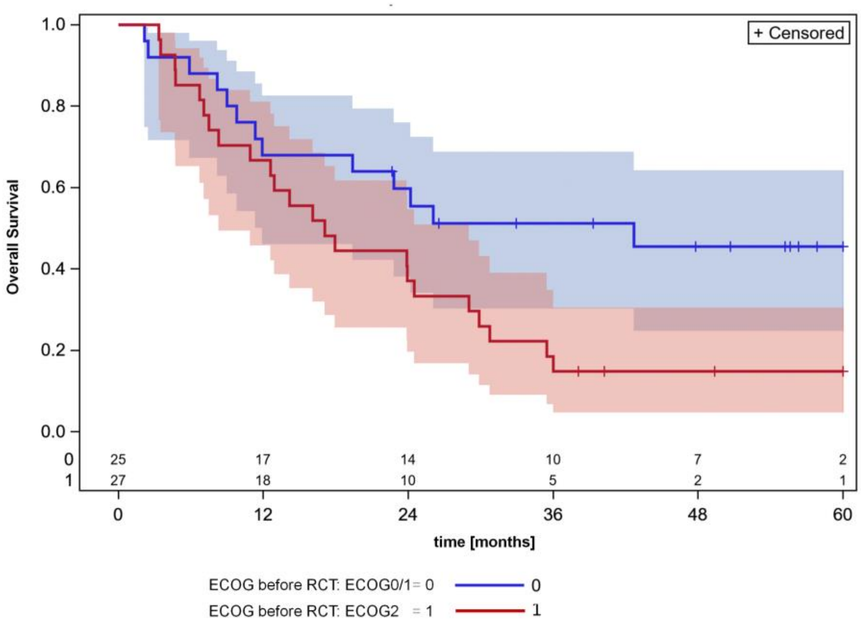

Figure S2 (b): : Overall Survival dependent on ECOG performance status: ECOG 0/1 [blue line 0] and ECOG 2 [red line 1].

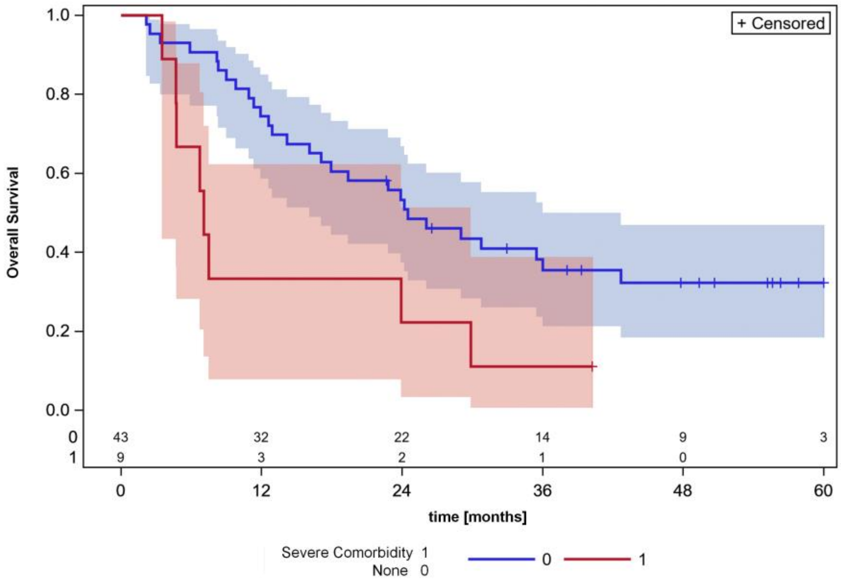

Figure S2 (c): : Overall Survival dependent on presence of severe comorbidity: no higher comorbidities [blue line 0] and severe comorbidities [red line 1].
